# The Refining Mechanism of Super Gravity on the Solidification Structure of Al-Cu Alloys

**DOI:** 10.3390/ma9121001

**Published:** 2016-12-10

**Authors:** Yuhou Yang, Bo Song, Zhanbing Yang, Gaoyang Song, Zeyun Cai, Zhancheng Guo

**Affiliations:** 1 School of Metallurgical and Ecological Engineering, University of Science and Technology Beijing, Beijing 100083, China; yyhyangyuhou@163.com (Y.Y.); yangzhanbing@ustb.edu.cn (Z.Y.); sgy_beike@163.com (G.S.); zexiaoyun@126.com (Z.C.); 2 State Key Laboratory of Advanced Metallurgy, University of Science and Technology Beijing, Beijing 100083, China; zcgustb@163.com

**Keywords:** super gravity, solidification structure, refining mechanism, Al-Cu alloy

## Abstract

There is far less study of the refining effect of super gravity fields on solidification structures of metals than of the effects of electrical currents, magnetic and ultrasonic fields. Moreover, the refining mechanisms of super gravity are far from clear. This study applied a super gravity field to Al-Cu alloys to investigate its effect on refining their structures and the mechanism of interaction. The experimental results showed that the solidification structure of Al-Cu alloys can be greatly refined by a super gravity field. The major refining effect was mainly achieved when super gravity was applied at the initial solidification stage; only slight refinement could be obtained towards the end of solidification. No refinement was obtained by the super gravity treatment on pure liquid or solid stages. The effectiveness of super gravity results from its promoting the multiplication of crystal nuclei, which we call “Heavy Crystal Rain”, thereby greatly strengthening the migration of crystal nuclei within the alloy. Increasing the solute Cu content can increase nucleation density and restrict the growth of crystals, which further increases the refining effect of super gravity. Within this paper, we also discuss the motile behavior of crystals in a field of super gravity.

## 1. Introduction

In studying the effect of external fields on the solidification structures of castings, many studies have investigated electric currents [1,2], magnetic [3] and ultrasonic fields [4,5,6]. EMS (electromagnetic stirring) technology in particular is widely used in steel plants. However, in centrifugal casting, most existing research has studied mold filling using a centrifugal method [7,8,9,10] and removal of impurities or recovery of valuable components [11,12]. In contrast, research on the solidification structure refinement using super gravity is relatively lacking. Although some researchers have looked into the effect of super gravity fields or centrifugal fields on refining the solidification of cast structures, the mechanism of grain refinement varies widely among these studies.

Chen et al. [13] found that the nucleation rate of metallic melt was increased by super gravity when they studied the change of nucleation energy in an elevated gravity field. Meanwhile, Zhao et al. [14] believed that changes to the nucleation rate of pure Al caused by Δ*G_b_* are not significant enough to achieve the desired structure refinement, and they attributed this to dendrite fragments caused by convection in a super gravity field. However, their study lacked a detailed explanation of how the super gravity generated that convection. Chang et al. [15] studied the microstructure evolution of Al alloys in centrifugal casting, and concluded that the dominant bulk nucleation mechanisms forming the equiaxed zone are attributable to the free-chill crystal theory and the dendrite fragments theory. However, they did not provide detailed analysis of the movement of crystal nuclei. Melgarejo et al. [16] found a grain size decrease in functionally-graded Al-Mg-B composites fabricated by centrifugal casting, and hypothesized that the decrease might be due to a faster solidification rate experienced in a centrifugal field as compared with that in normal gravity casting, but their paper did not devote much discussion to this possibility. In Wu’s research [17] on the numerical simulation of the microstructure evolution of a Ti-6Al-4V alloy in vertical centrifugal casting, a larger cooling rate was obtained due to the recommended wetting of the mold in centrifugal casting. Unfortunately, this study did not address the floating or settling of equiaxed grains. In summary, existing research hypotheses on the structure refinement mechanism of metals in a super gravity field or centrifugal field are limited to existing research methods. Since different mechanisms may dominate under different conditions, the mechanism of grain refinement remains unclear; hence a systematical investigation is urgently needed.

In our research, we obtained significant structure refinement of Al-Cu alloys in a super gravity field, and what we call “Heavy Crystal Rain” was identified as the refining mechanism. This is because super gravity greatly promotes the moving of crystal nuclei, causing grain multiplication and refining the final solidification structure.

## 2. Materials and Methods

### 2.1. Experimental Equipment

**Figure 1:**
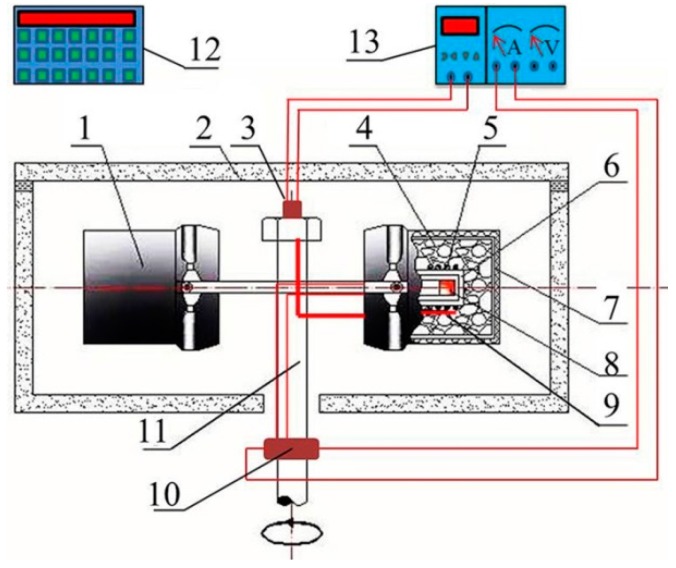
Schematic diagram of the experimental super gravity apparatus: 1. counterpart; 2. housing; 3. thermal couple conductive slip ring; 4. thermal insulation materials; 5. resistance wire; 6. furnace tube; 7. furnace shell; 8. sample; 9. thermal couple; 10. conductive slip ring; 11. rotation axis; 12. rotation controller; 13. temperature controller.

In this research, a super gravity field was generated by centrifugal rotation of the experimental super gravity apparatus. The setup, schematically shown in Figure 1 [18], mainly consists of a rotating system with two furnaces symmetrically installed onto a horizontal rotor and a heating system controlled by a program controller within the observed precision range of ±3 K (±3 °C) by an R type thermocouple. The super gravity field was evaluated using the gravity coefficient, which is defined as the ratio of centrifugal acceleration to the normal acceleration via Equation (1).

(1) $$G=\sqrt{g^{2}+{\left(\omega^{2}r\right)}^{2}}/g=\sqrt{g^{2}+{\left(\frac{N^{2}\pi^{2}r}{900}\right)}^{2}}/g$$

where *G* is the gravity coefficient; ω is the rotational angular velocity, rad·s^−1^; *N* is rotating speed, r·min^−1^; R is the distance between the rotation center and the sample, 0.25 m; g is the normal gravity acceleration, 9.8 m·s^−2^.

### 2.2. Materials and Procedures

The Al-Cu binary alloy for our super gravity experiments was manufactured from pure Al and pure Cu. Pure Al was melted at 800 °C with argon gas protection in a molybdenum wire furnace, then a specific mass of pure Cu was added into the melt and stirred every 10 min to ensure composition uniformity. The molten alloy was then water quenched after heat preservation for 1 hour at 800 °C. A basic alloy was obtained in this process. A mixture of 33 wt % sodium chloride and 67 wt % calcium chloride was used as covering slag to protect the samples from oxidation during the experiments.

In our experiment, the basic alloy (48 g) and covering slag (5 g) were put into a graphite crucible (inner diameter 21 mm) to be heated to 750 °C in the heating furnace. After holding at 750 °C for 10 min, the melt was cooled down at a cooling rate of 10 °C/min and the centrifuge apparatus was started and adjusted to the target angular velocity to apply the super gravity field when temperature fell to the liquidus temperature. The apparatus was not shut off until temperature fell to 50 °C lower than the solidus temperature to ensure complete solidification. Then the sample was water quenched to maintain its solidification structure. Simultaneously, a parallel sample was treated in a normal gravity field under the same cooling conditions. Each sample was sectioned longitudinally into two halves along the central plane, one of which was burnished and polished for metallographic examination. The etching reactant used for revealing the solidification structure was a mixture of 6 parts nitric acid: 2 parts hydrofluoric acid: 92 parts water. The macrostructure and the microstructure of the resulting solids were examined by a digital camera and an optical microscope (9XB-PC type), respectively.

## 3. Results

### 3.1. Macro- and Micro-Structure of Al-4.5 wt % Cu Alloy in Normal Gravity and Super Gravity Fields

**Figure 2:**
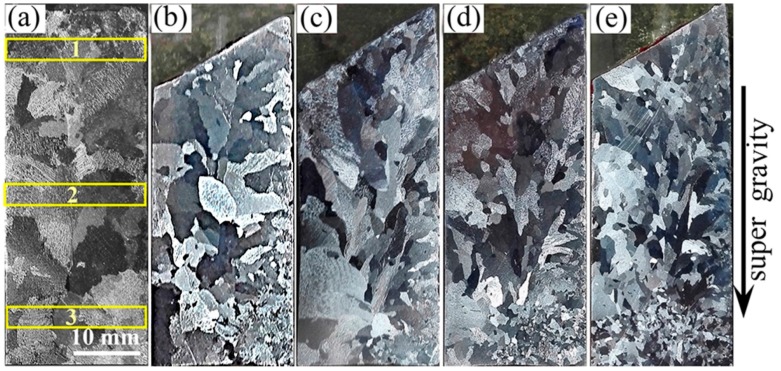
Macrostructures of Al-4.5 wt % Cu alloy in normal gravity field and super gravity field at the cooling rate of 10 °C/min. (**a**) *G* = 1; (**b**) *G* = 100; (**c**) *G* = 300; (**d**) *G* = 500; (**e**) *G* = 800.

Figure 2 shows the macrostructure of the Al-4.5 wt % Cu alloys obtained in a normal gravity field and a super gravity field at the cooling rate of 10 °C/min. When in a normal gravity field, as exhibited in Figure 2a, the solidification structure is composed of coarse columnar and bulk crystals. While in a super gravity field of *G* = 100, significant refinement of the solidification structure is observed, especially at the latter half of the sample, though some large grains still exist at the first half as shown in Figure 2b. The structure becomes further refined as the gravity coefficient is increased, as shown in Figure 2c,d, with some very fine crystals appearing at the bottom of the image. When the gravity coefficient is increased up to 800, very fine grains occupy the whole sample, particularly towards the bottom of the sample, without any of the large grains seen in the sample created in the normal gravity field.

**Figure 3:**
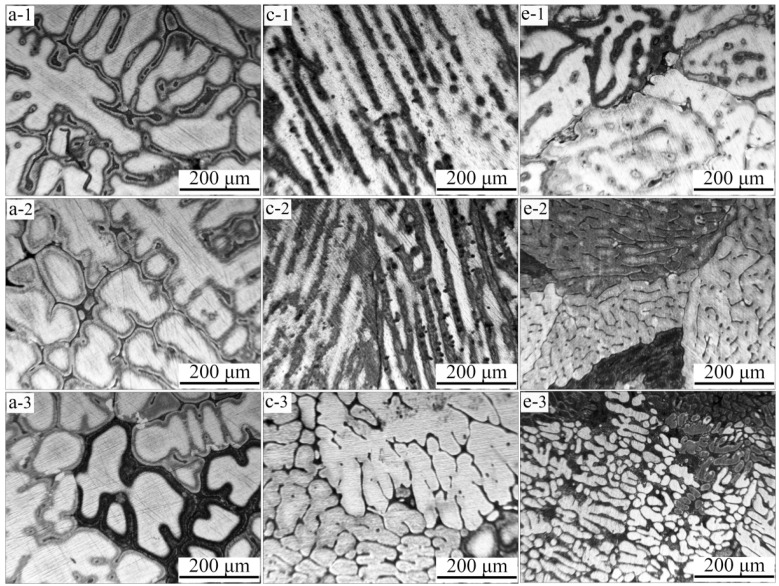
Microstructure of Al-4.5 wt % Cu alloy at different positions of samples in normal gravity and super gravity field: (**a-1**–**a-3**) *G* = 1; (**c-1**–**c-3**) *G* = 300; (**e-1**–**e-3**) *G* = 800. ((1) through (3)) refer to positions 1 through 3, respectively, marked in Figure 2a.

Because the macrostructure in the samples varies greatly at different positions, three areas were selected for microstructure observation of the longitudinal section of the sample as depicted in Figure 2a. The results obtained with gravity coefficients of *G* = 1, 300 and 800, corresponding to the macrostructure in Figure 2a,c,e, are shown in Figure 3. In a normal gravity field, developed dendritic crystals dominate all three areas. When the gravity coefficient is increased to 300, a lathy microstructure dominates the top and middle areas while the bottom area exhibits a dendrite structure, which is much finer than that under a normal gravity field. When the gravity coefficient is increased to 800, extremely fine dendritic grains are seen in all three areas. Compared vertically, the dendrites become finer and shorter approaching the bottom of the sample, which is consistent with the overall macrostructure. In general, the dendrite size decreases as the gravity coefficient is increased, indicating that super gravity can greatly refine the solidification structure of Al-4.5 wt % Cu alloy.

### 3.2. The Effect of Solute Content on the Solidification Structure Refinement by Super Gravity

**Figure 4:**
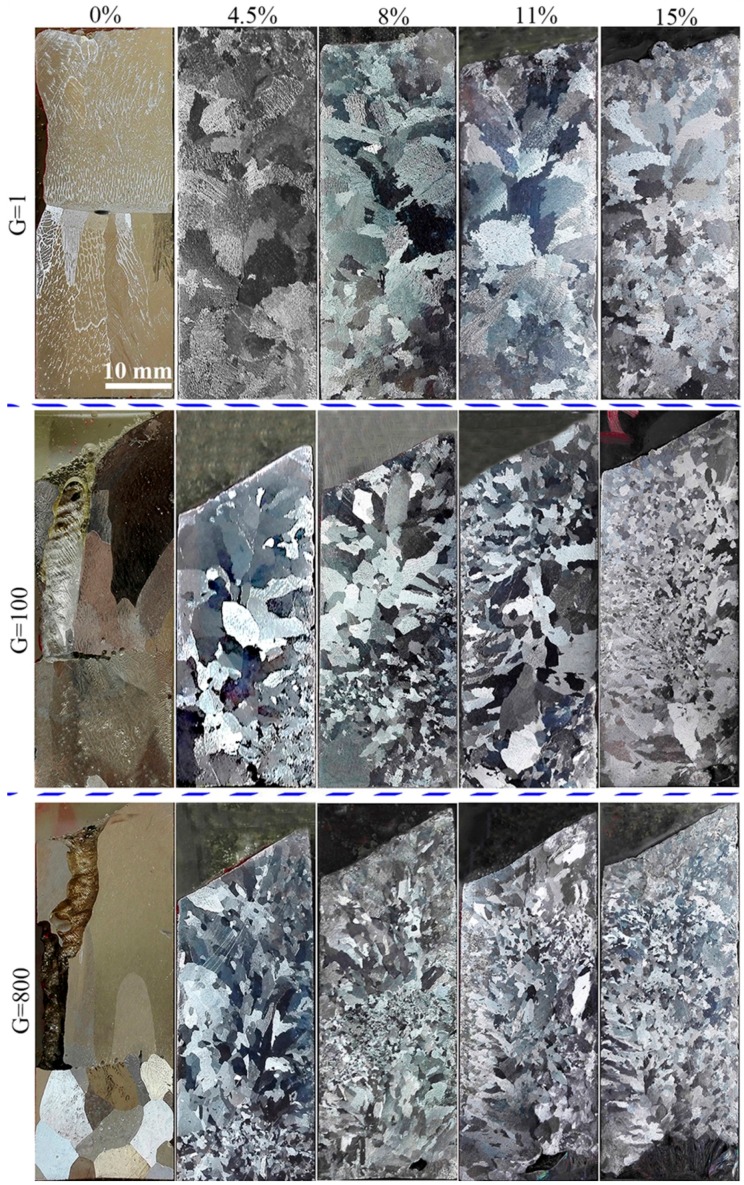
Macrostructure of Al-Cu alloys with varying solute contents in normal gravity field and super gravity field: the subfigures in the first row correspond to samples in a normal gravity field; the subfigures in the second row and the third row correspond to samples in super gravity fields of G = 100 and G = 800, respectively.

In order to investigate the effects of alloy composition on refining alloy solidification structures using super gravity, experiments were carried out using five different alloys with Cu weight percentages of 0, 4.5, 8, 11, and 15, respectively. Experiments were carried out in a normal gravity field and a super gravity field of *G* = 100 and *G* = 800. Figure 4 shows the macrostructures of Al-Cu alloys with varying Cu contents in normal and super gravity fields. As shown in the first row, when in a normal gravity field, no obvious grain morphology can be seen in pure Al, as the grain size is too large. When the Cu content increases to 4.5 wt %, the structure exhibits coarse columnar grains compared with that of pure Al. The structure gets more refined with increased Cu content, but continues to exhibit coarse columnar and bulk grains. When in a super gravity field at *G* = 100, the macrostructure of pure Al resembles that in the normal gravity field. However, as the Cu content increases to 4.5 wt %, much finer grains appear, although some bulk crystals remain. The grain size decreased markedly with increased Cu content, and when the Cu content is 15 wt %, fine globular grains dominate most of the sample. When in a super gravity field of *G* = 800, the solidification structure of pure Al is much finer than that in a normal gravity field or a small super gravity field of *G* = 100, with several equiaxed grains at the bottom of the sample. All the Al-Cu alloys show much finer solidification structures as the gravity coefficient increases up to 800; additionally, the grain size becomes smaller with the increasing Cu content.

It is noteworthy that with a gravity coefficient of *G* = 800, a fine grain zone (FGZ) can be found in the Al-Cu alloys. When the Cu content is 4.5 wt %, the FGZ appears at the bottom of the sample. With increasing Cu content, the FGZ moves upward, its area increases, and grain size decreases. When Cu content is increased to 15 wt %, the FGZ occupies most of the sample.

### 3.3. Macrostructure of Applying Super Gravity at Different Solidification Stages of Al-8 wt % Cu Alloy

**Figure 5:**
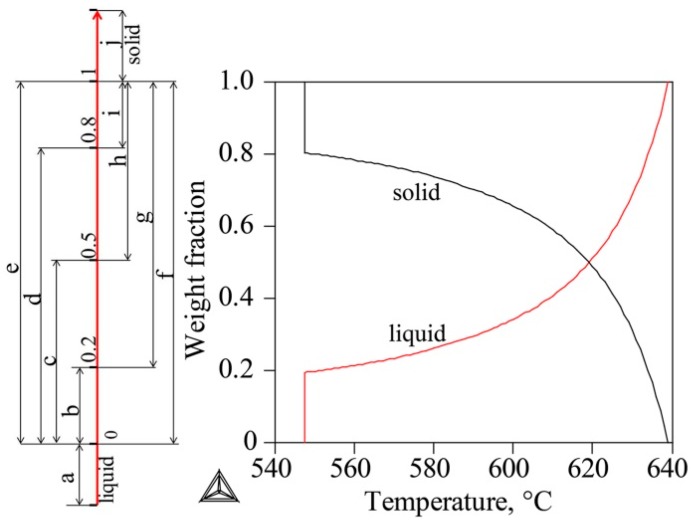
The solidification process of Al-8 wt % Cu alloy calculated via Thermo-Calc software using the Scheil mode.

In order to clarify the refining mechanism of super gravity on the solidification structure of the Al-Cu alloy, super gravity of *G* = 800 was applied at different solidification stages of Al-8 wt % Cu alloy as shown in Figure 5, which charts the solid and liquid fraction change calculated via Thermo-Calc software using the Scheil-mode.

**Figure 6:**
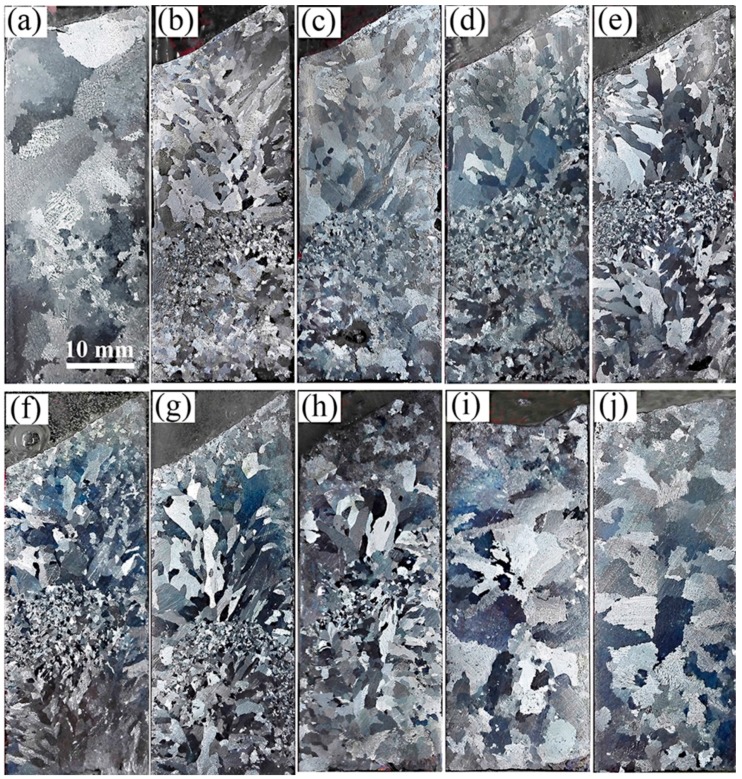
Macrostructures of Al-8 wt % Cu alloy after applying a super gravity field of *G* = 800 at different solidification stages as shown in Figure 5 (**a**–**j**) corresponds to super gravity treatment at a–j stages shown in Figure 5.

As shown in Figure 6a,j, when applying a super gravity field at the high-temperature liquid stage or the completely solidified stage, the solidification structures exhibit coarse columnar and bulk grains like those seen in the structures not treated with super gravity, demonstrating that super gravity cannot refine the solidification structure of the complete liquid stage or of the solidified metallic alloy. Super gravity cannot create an inoculation effect for refining the solidification structure of liquid metal.

Figure 6b–e correspond to super gravity treatment at the b–e stages of Figure 5. The super gravity treatment was always started at the onset of solidification and ended when the solid fraction reached 20 wt %, 50 wt %, 80 wt % and 100 wt %, respectively. All the solidification structures seen are fine globular or columnar grains, and prolonging treatment gradually increases the visible area of fine grain zone (FGZ). This shows that the solidification structure of Al-8 wt % Cu alloy can be effectively refined by applying super gravity at the initial solidification stage.

Figure 6f–i show the results of super gravity treatment at the f–i stages of Figure 5, with super gravity treatment started once the solid fraction reached 0 wt %, 20 wt %, 50 wt %, 80 wt %, respectively, and ended when solid fraction reached 100 wt %. By delaying the super gravity treatment, the refining effect of super gravity gradually fades away. In particular, the area of FGZ decreased significantly. When super gravity treatment is delayed until the solid fraction of the alloy reaches 80 wt %, refinement almost disappears and the solidification structure resembles that without super gravity treatment.

**Figure 7:**
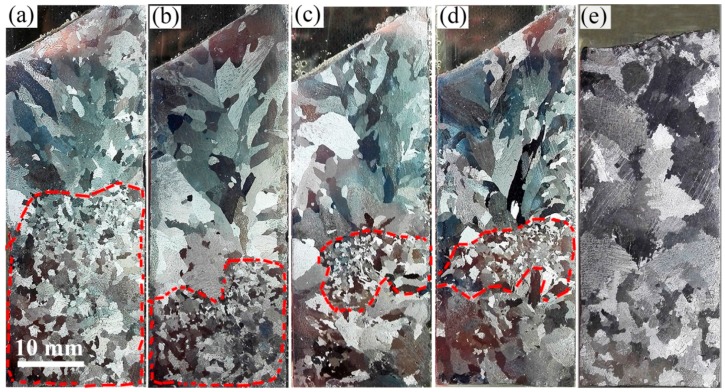
Macrostructures of Al-8 wt % Cu alloy after applying super gravity at different solidification stages by *G* = 300, ν = 5 °C/min. (**a**–**d**) correspond to super gravity treatment at the (**b**–**e**) stages of Figure 5.

In order to clarify the movement and distribution of crystals during the solidification process, experiments were designed for applying a relatively small super gravity field of *G* = 300 at a relatively low cooling rate of ν = 5 °C/min at solidification stages of b, c, d, and e as shown in Figure 5. Figure 7 shows the results, with the fine grain zone (FGZ) of each sample indicated by a red dashed line. Figure 7a corresponds to super gravity treatment at stage (b) in Figure 5. The structures in the lower half of the sample are fine globular crystals, but the upper section contains mainly coarse columnar crystals. Figure 7b corresponds to super gravity treatment at stage (c) of Figure 5. The FGZ is limited to the bottom third of the sample and the grain size in this area is decreased compared to that of Figure 7a. Figure 7c corresponds to super gravity treatment at stage (d) of Figure 5. Interestingly, the FGZ moves upward to nearly the middle of the sample and the grain size in this area becomes even finer. Figure 7d corresponds to super gravity treatment at stage (e) of Figure 5. The solidification structure here is almost identical to the structure in Figure 7c except that the area of FGZ extends a bit.

A parallel sample without super gravity treatment at ν = 5 °C/min was also obtained. Shown in Figure 7e, the structure of this sample is mainly composed of large bulk grains with some relatively small crystals at the bottom area of the sample.

## 4. Discussion

Previous research shows that one of the most important characteristics of super gravity is its extreme intensification of relative movement between solid and liquid (S-L) or liquid and liquid (L-L) stages, as long as density difference exists in the system [18,19,20,21,22,23,24]. Hence for this study we focus on the crystals’ movement and distribution relative to the density change from solid to liquid, which has a close relationship with grain refinement in a super gravity field.

### 4.1. Density Change of Primary α-Al Crystal and Liquid Phases in Al-Cu Alloys during Solidification

**Figure 8:**
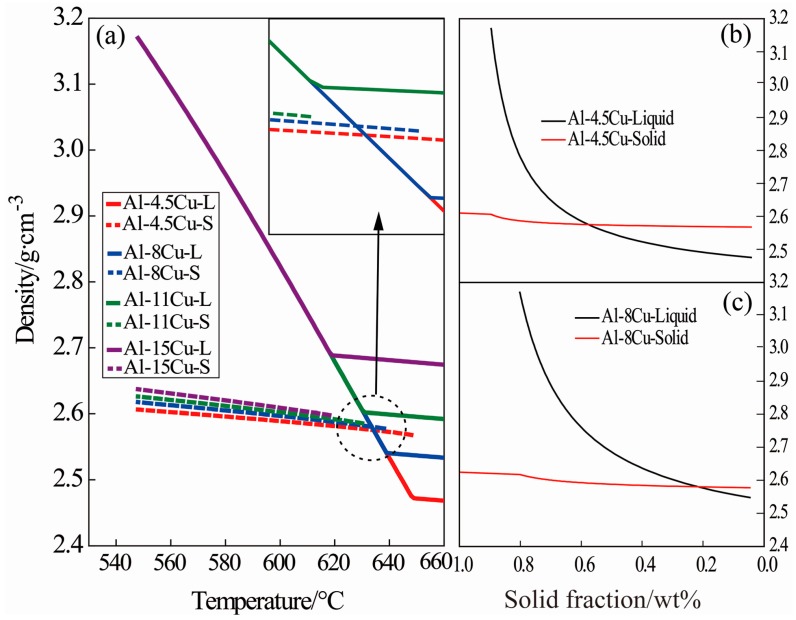
Density change of solid and liquid phase in alloys during solidification calculated using Jmatpro7.0 software. (**a**) density change with temperature; (**b**,**c**) density change with solid fraction of Al-4.5 wt % Cu and Al-8 wt % Cu alloys, respectively.

**Figure 9:**
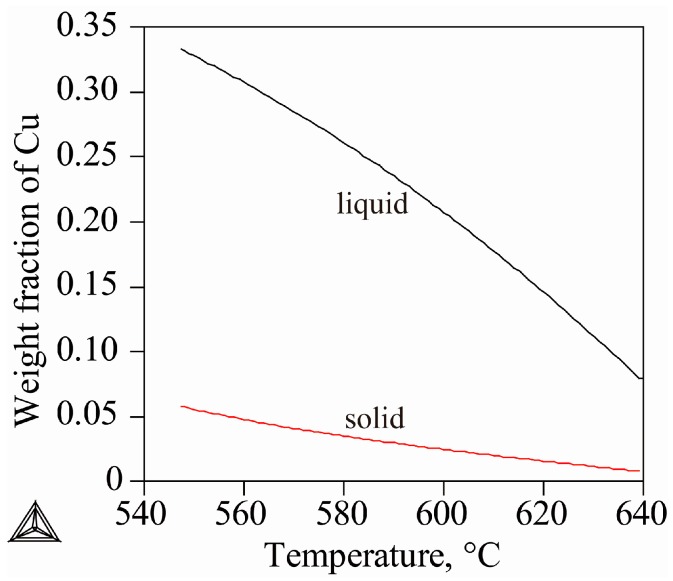
The distribution of solute Cu in solid and liquid phases of Al-8 wt % Cu alloy during solidification, calculated by Thermal-Calc software.

Because of the wide solid-liquid zone of Al-Cu alloy, the sample will not completely solidify quickly under test conditions (a cooling rate of ν = 10 °C/min). The densities of the primary α-Al crystals and the remaining liquid are in flux throughout the solidification period. Figure 8 shows the density change of solid and liquid phase of Al-Cu alloys throughout the solidification process as calculated using Jmatpro7.0 software. Figure 8a shows that the density of primary α-Al crystals increases slightly, but that of the remaining liquid increases rapidly with decreasing temperature. The density increase of the solid α-Al and the remaining liquid could be due to the temperature decline and the enrichment of solute Cu [25], which is shown in Figure 9. Temperature and enrichment were calculated using Thermal-Calc software in the Scheil mode, under the assumption that no solute diffusion exists in the solid while completely uniform diffusion is in the liquid phase. In fact, the density of liquid varies as a function of both temperature and solute content. Before solidification starts, the density of liquid metal increases quite slowly, so that that temperature decline cannot lead to a rapid density increase of liquid. When solidification starts, saltation appears for the density change, the result of rapid enrichment of solute Cu in the liquid as shown in Figure 9 for the small partition coefficient of Al-Cu alloy [26,27]. It is known that the density of Al-Cu alloy will generally increase with increasing Cu content, as the density of Cu is much larger than that of Al.

At the initial solidification period, the density of primary α-Al is greater than that of the liquid for Al-4.5 wt % Cu and Al-8 wt % Cu alloys. However, because of the rapid enrichment of solute Cu in the liquid, the liquid density will exceed that of solid α-Al at a certain temperature. But the density of the primary solid α-Al for Al-11 wt % Cu and Al-15 wt % Cu alloys is always less than that of the corresponding liquid throughout the solidification process.

Figure 8b,c show the density changes of Al-4.5 wt % Cu alloy and Al-8 wt % Cu alloys, respectively, demonstrating that when the density of liquid exceeds that of the solid α-Al, the solid component of the sample reaches approximately 59.18 wt % and 23.16 wt %, respectively, which is crucial to the movement and distribution of crystals in a super gravity field.

### 4.2. The “Heavy Crystal Rain” Mechanism in a Super Gravity Field

Zhao et al. [14] studied the grain refinement of pure Al using super gravity, hypothesizing that the main reason for grain refinement was the dendrite fragments caused by the turbulent flow of a super gravity field providing more nuclei in the melting stage. However, their study only showed the microstructure at the center of the sample without providing the macrostructure of the whole sample. This is a significant oversight, since the crystal movement in a super gravity field has a prominent influence on the solidification structure. Furthermore, their study showed no strong evidence of the dendrite fragmentation mechanism. We believe it is unlikely that excess nuclei give rise to dendritic morphology and are then broken up into fragments under the effect of turbulent convection. Moreover, there are crucial differences between our study of solidification in a super gravity field and the convectional centrifugal casting. In a convectional centrifugal casting process [15,28], the chilling grain region appears just as the molten metal is poured into the mold. With centrifugal rotation, the region of solid-liquid interface experiences turbulent flow due to abrupt velocity change at the interface. This agitation of the chilling grains may break off some dendrites and disperse them in the melt, causing some solidification structure refinement during the casting. In the present study, the metal experienced no chilling effect since it was melted and solidified in the super gravity furnace at a controlled cooling rate without an abrupt velocity change and without experiencing turbulent flow. Hence, the dendrite fragment hypothesis does not apply.

The “crystal rain” we observed early in the solidification process is believed to be the main mechanism of structural refinement of pure Al through electrical current pulse and pulse magneto-oscillation seen in other experiments [29,30]. In our opinion, the experiments by Liao et al. and Gong et al. were well-designed. In that work, treatment at the nucleation stage had the best refining effect, while the solidification structures were slightly refined with treatment at the beginning and end of crystal growth, which corresponds to our results.

It is a fact that solids will float upward in liquid if the density of the solid is less than that of the liquid, while a solid will sink if its density is greater than that of the liquid. In our experiment, the viscosity of the melt and the small size of the primary α-Al nuclei inhibit movement of the crystal nuclei within the melt during the solidification process. Therefore, obvious floatation or sedimentation of crystals will not be observed in a normal gravity field. However, obvious crystal floatation or sedimentation can be generated using super gravity. The motion of a spherical solid particle in viscous liquid can be expressed according to Stokes’ law [31,32]:

(2) $$\frac{\pi }{6}d^{3}(\rho_{l}-\rho_{p})Gg-3\pi \eta d\frac{dr}{dt}=\frac{\pi }{6}d^{3}\rho_{p}\frac{d^{2}r}{dt^{2}}$$

where *d* is the diameter of the particle m; *ρ_l_* and *ρ_p_* are the densities of the liquid and solid particle kg·m^−3^; *η* is the dynamic viscosity of the liquid Pa·s; and r is the displacement of the particle m.

When the particle’s movement reaches equilibrium, the right side of Equation (2) equals zero, and the particle’s moving velocity can be expressed as:

(3) $$V_{r}=d^{2}(\rho_{l}-\rho_{p})Gg/18\eta$$

where *V_r_* is the particle’s moving velocity in the viscous liquid.

The particle’s moving velocity in equilibrium is in direct proportion to the square of its diameter and gravity coefficient according to Equation (3). In terms of the present study, when the Al-4.5 wt % Cu alloy, for instance, was solidified in the super gravity field, since the density of the primary α-Al nuclei is larger than that of the remaining liquid in the early solidification stage as seen in Figure 8b, obvious sedimentation of crystals occurs as the settling velocity of the crystal nuclei is greatly increased by super gravity. This is what we call the “Heavy Crystal Rain”, and it leads to the multiplication of crystals at the bottom of the sample. Although the crystals should float upward at the latter solidification stage, since the density of the remaining liquid exceeds that of the solid, the significant fraction of solid prevents their free movement. As a result, the solidification structure at the bottom of the sample is finer than that of the top area, which is consistent with the experimental result shown in Figure 2. This can be further seen by examining the microstructure variations of grain size within the sample in Figure 3. When *G* = 300, the dendrite size at the bottom is decreased due to the accumulation of fallen crystal nuclei. The lathy dendrites at the upper and middle areas of the samples may be the result of the effect of super gravity on the growth direction of the dendrites and plastic deformation. When *G* = 800, the “crystal rain” is too heavy, so that the dendrites in all three areas cannot rise, change growth direction and cause deformation. Thus, the resulting microstructures are all fine dendrites, though the sample still exhibits variations in grain size. Moreover, the “crystal rain” occurs during the first half of solidification as shown by the results in Figure 6. This is because during early solidification, the crystals can move freely, but in the second half of the solidification process, the fraction of solid is too large, and the dendrite connections too established, for the crystals to move freely [25]. Hence the structural refinement gets worse by delaying super gravity treatment, as revealed by the decreasing FGZ in Figure 6f–j.

According to the observed “heavy crystal rain” phenomenon, the formation of FGZ depends on motion and distribution of crystal nuclei that can be explained by the density changes of solid and liquid. For Al-4.5 wt % Cu alloy, with a gravity coefficient of *G* = 800, the crystals moved to the bottom of the sample before the solid fraction reached 59.13 wt %; thereafter, the crystals should rise within the sample according to the density change in Figure 8, but by that point the solid fraction large enough to prevent the crystals from moving freely [25] and we see the FGZ appeared at the bottom of the sample. For an Al-8 wt % Cu alloy with a gravity coefficient of *G* = 800, the crystal nuclei move to the bottom of the sample before the solid fraction reached 23.16 wt %; thereafter the crystals rise according to Figure 8c. As the fraction of solid increases, the crystals accumulate and the FGZ appears at the middle of the sample. For Al-11 wt % Cu and Al-15 wt % Cu alloys with a gravity coefficient of *G* = 800, the density of the crystals is always less than that of the liquid, so the crystals rise throughout the process, causing the FGZ to occupy the top of the samples. It can also be seen that the solidification structure is more refined with increasing solute Cu content, as shown in Figure 4. As super gravity has the best refining effect at the initial solidification period, structural refinement by super gravity must be closely related to the nucleation of the Al-Cu alloy. This is because, under any conditions, the nucleation density at high concentrations is larger than that for low concentrations, and the dendrite growth velocity is much faster for low-concentration alloys [15,33,34]. As more nuclei are formed, the “heavy crystal rain” is heavier in a super gravity field. Therefore, solidification structure becomes finer by increasing the Cu content in a super gravity field.

Based on the experimental results shown in Figure 7, we can better understand how the crystals move and are distributed during the solidification process of an Al-8 wt % Cu alloy under the effect of super gravity at *G* = 300. During the initial solidification stage, as the first batch of nuclei form, they move downward quickly under the force of super gravity even as they form crystals. When the fraction of solid in the sample reaches 20 wt %, super gravity treatment ends and the crystals are distributed through the second half of the sample, causing grains to multiply in this area. As a result, the grain size in the second (lower) half of the sample is finer than that at the top, as shown in Figure 7a. In the sample for which the super gravity field ends at a solid fraction as it reaches 50 wt %, although the density of the liquid exceeds that of the solid, the crystals’ direction of movement changes very slowly, so that the crystals keep on moving to the bottom of the sample. Hence the crystals cause the FGZ at the bottom of the sample, as shown in Figure 7b. With further treatment, ending when the solid fraction reaches 80 wt %, the crystals reverse direction and began to move upward within the sample, gathering in the middle of the sample as shown in Figure 7c. When super gravity is applied throughout the solidification process, the crystals collect in the middle of the sample, shown in Figure 7d. In a normal gravity field, the crystals also settle [25,35,36]. In our experimental result shown in Figure 7e, the solidification structure at the bottom area is obviously finer than that at the top of the sample, a difference caused by the settling of the crystals. However, the crystal sedimentation is far less than that formed by a super gravity field, so that the grain size in the FGZ is much larger than that formed in the super gravity field.

Although the “heavy crystal rain” is the main mechanism of structural refinement in a super gravity field, we must also point out that, in addition to the effects of the gravity coefficient and the respective densities of solid and liquid, both crystal growth and the viscosity change of the melt during the solidification process also influence the moving behavior of crystals, according to Stokes’ law, and this too influences the final solidification structure.

Although super gravity can be used to refine the alloy solidification structure, it also causes structural heterogeneity in the casting. This homogeneity negatively affects the cast solids’ mechanical properties; however, this effect can be improved and even eliminated in follow-up processes such as heat treatment and rolling. Indeed, the solidification structure of the current method of continuous castings is not uniform either, and the results are also subject to the rolling process. This “heterogeneity” brought about by super gravity can be beneficial to the fabrication of functionally graded materials, as described in references [16,20,21].

## 5. Conclusions

(1) The solidification structure of Al-Cu alloys can be greatly refined using super gravity. The refining effect increases with both the increase of the gravity coefficient and increased Cu content in the solute.
(2) The main refining effect occurs when super gravity is applied at the initial solidification stage. Only slight refinement can be obtained during the second half of the solidification process. The structure cannot be refined by super gravity treatment while the alloy is completely liquid and solid metals.
(3) The structural refining mechanism of an Al-Cu alloy using super gravity is the result of super gravity’s effect in greatly strengthening the settling and floating of the crystal nuclei and promoting the multiplication of crystal nuclei. Increasing the solute Cu content also increases the nucleation density, and restricts the growth of solid crystals, both of which can further increase the refining effect of super gravity.
